# Activity of Scorpion Venom-Derived Antifungal Peptides against Planktonic Cells of *Candida* spp. and *Cryptococcus neoformans* and *Candida albicans* Biofilms

**DOI:** 10.3389/fmicb.2016.01844

**Published:** 2016-11-18

**Authors:** Fernanda Guilhelmelli, Nathália Vilela, Karina S. Smidt, Marco A. de Oliveira, Alice da Cunha Morales Álvares, Maria C. L. Rigonatto, Pedro H. da Silva Costa, Aldo H. Tavares, Sônia M. de Freitas, André M. Nicola, Octávio L. Franco, Lorena da Silveira Derengowski, Elisabeth F. Schwartz, Márcia R. Mortari, Anamélia L. Bocca, Patrícia Albuquerque, Ildinete Silva-Pereira

**Affiliations:** ^1^Laboratory of Molecular Biology, Department of Cellular Biology, Institute of Biological Sciences, University of BrasíliaBrasília, Brazil; ^2^Laboratory of Applied Immunology, Department of Cellular Biology, Institute of Biological Sciences, University of BrasíliaBrasília, Brazil; ^3^Laboratory of Molecular Biophysics, Department of Cellular Biology, Institute of Biological Sciences, University of BrasíliaBrasília, Brazil; ^4^Faculty of Medicine, University of BrasíliaBrasília, Brazil; ^5^Center of Proteomic and Biochemistry Analysis, Post Graduation in Biotechnology and Genomic Sciences, Catholic University of BrasíliaBrasília, Brazil; ^6^Laboratory of Neuropharmacology, Department of Physiological Sciences, Institute of Biological Sciences, University of BrasíliaBrasília, Brazil; ^7^Faculty of Ceilândia, University of BrasíliaBrasília, Brazil

**Keywords:** antifungal drugs, antimicrobial peptides, *Candida* spp., *Candida albicans*, *Cryptococcus neoformans*, scorpions venom

## Abstract

The incidence of fungal infections has been increasing in the last decades, while the number of available antifungal classes remains the same. The natural and acquired resistance of some fungal species to available therapies, associated with the high toxicity of these drugs on the present scenario and makes an imperative of the search for new, more efficient and less toxic therapeutic choices. Antimicrobial peptides (AMPs) are a potential class of antimicrobial drugs consisting of evolutionarily conserved multifunctional molecules with both microbicidal and immunomodulatory properties being part of the innate immune response of diverse organisms. In this study, we evaluated 11 scorpion-venom derived non-disulfide-bridged peptides against *Cryptococcus neoformans* and *Candida* spp., which are important human pathogens. Seven of them, including two novel molecules, showed activity against both genera with minimum inhibitory concentration values ranging from 3.12 to 200 μM and an analogous activity against *Candida albicans* biofilms. Most of the peptides presented low hemolytic and cytotoxic activity against mammalian cells. Modifications in the primary peptide sequence, as revealed by *in silico* and circular dichroism analyses of the most promising peptides, underscored the importance of cationicity for their antimicrobial activity as well as the amphipathicity of these molecules and their tendency to form alpha helices. This is the first report of scorpion-derived AMPs against *C. neoformans* and our results underline the potential of scorpion venom as a source of antimicrobials. Further characterization of their mechanism of action, followed by molecular optimization to decrease their cytotoxicity and increase antimicrobial activity, is needed to fully clarify their real potential as antifungals.

## Introduction

An increase of systemic mycoses incidence due to the rising number of immunocompromised individuals, particularly cancer, HIV/AIDS and solid-organ transplant patients has been noticed in recent years. *Candida* spp. and *Cryptococcus* spp. are among the main causative agents (Romani, 2011; Armstrong-James et al., 2014).

*Candida* spp. remain the most important cause of opportunistic mycoses worldwide and are associated to high mortality and morbidity rates, *Candida albicans* being the major agent of candidemia (Pfaller and Diekema, 2007; Mikulska et al., 2012). Furthermore, an increase in infections caused by non-albicans species has been noticed, like *Candida parapsilosis*, *Candida tropicalis*, and *Candida glabrata*, the latter being more resistant to antifungal drugs than other *Candida* species (Sobel, 2006; Pfaller and Diekema, 2007; Diekema et al., 2012; Rodrigues et al., 2014). *Cryptococcus neoformans* is another opportunistic pathogen of clinical relevance, especially among HIV/AIDS patients. Cryptococcal meningitis is the most important cause of death from HIV-related fungal infection worldwide (Park et al., 2009). The estimated yearly incidence of cryptococcal meningitis is almost one million cases, causing 625,000 deaths (Park et al., 2009). In addition, *Candida* species and *C. neoformans* can form biofilms on abiotic and biotic surfaces (Mayer et al., 2013; Martinez and Casadevall, 2015). Of particular interest is the formation of biofilms by *C. albicans*, which confers specialized properties to the microorganism that complicate treatment, such as the increased resistance to antimicrobial drugs and the ability to evade the host immune system, among others (Finkel and Mitchell, 2011; Fanning and Mitchell, 2012). Another problem regarding fungal infections is the rising of resistance to currently available antifungal drugs (Pan et al., 2012; Pfaller, 2012; Arendrup, 2013). This is compounded by the intrinsic resistance of some fungi to antifungals, such as *C. neoformans* to echinocandins and *C. glabrata* to azoles (Sable et al., 2008; Pfaller, 2012; Paul and Moye-Rowley, 2014).

In this scenario, there is a concrete need for new antifungals, and antimicrobial peptides (AMPs) have been promoted as promising in that regard (Hancock and Sahl, 2006), considering their low molecular masses and lower potential to induce resistance (Mookherjee and Hancock, 2007; Huang et al., 2010).

A great number of peptides are present in scorpion venom. They can be classified as disulfide-bridged peptides or non-disulfide-bridged peptides (NDBPs), the latter being the major component of their venom (Almaaytah and Albalas, 2014; Harrison et al., 2014; Ortiz et al., 2015). Most have similar physicochemical characteristics, such as a cationic character and structural flexibility. NDBPs present diverse biological activities, including antimicrobial, cytolytic, immunomodulatory, antitumor, and bradykinin-like potentiation effects (Zeng et al., 2005; Almaaytah and Albalas, 2014; Ortiz et al., 2015). In the present work, we evaluated the antifungal activity of peptides previously obtained by transcriptomic approaches from venom glands of three scorpion species (Silva et al., 2009). We classified them according to the rules proposed by Zeng et al. (2005), who group scorpion NDBPs in six subfamilies based on their pharmacological action, length and structural similarity. The peptides presented here belong to subfamilies 3, 4, and 5 and their activity will be discussed following this classification. Some AMPs are presented here for the first time, among them five peptides from the venom of the scorpion *Tityus obscurus* corresponding to the first AMPs characterized in this species.

## Materials and Methods

### cDNAs Encoding Putative Antimicrobial Peptides

Different cDNAs encoding putative AMPs from the venom gland of scorpions *T. obscurus*, *Tityus costatus*, *Hadrurus gertschi*, and *Opisthacanthus cayaporum* were characterized by expressed sequence tag (EST). The amino acid sequences deduced from ESTs as described in literature (Diego-Garcia et al., 2005; Schwartz et al., 2007; Silva et al., 2009; Guerrero-Vargas et al., 2012) were considered to obtain the synthetic peptides as specified bellow (**Table 1**).

**Table 1:** Sequence of chemically synthesized peptides obtained from scorpion venom cDNA library sequencing.

Peptide	Subfamily	UniProt entry	Sequence	Species
ToAP2	3	LT576030	FFGTLFKLGSKLIPGVMKLFSKKKER	*T. obscurus*
ToAP2S1	3		FFGTLFKLLSKLIPGLMKLFSKLLER-NH_2_	Modified
Con10	3	C5J897	FWSFLVKAASKILPSLIGGGDDNKSSS	*O. cayaporum*
ToAP3	4	^∗^	FIGMIPGLIGGLISAIK-NH2	*T. obscurus*
NDBP-4.23	4	Q5G8B5/S6D3A7	FLGMIPGLIGGLISAFK-NH_2_	*T. costatus*; *T. obscurus*
ToAP1	4	LT576029	FIGMIPGLIGGLISAFK-NH_2_	*T. obscurus*
ToAP4	4	^∗^	FFSLIPSLIGGLVSAIK-NH2	*T. obscurus*
NDBP-5.6	5	P0C8W2	FIFDLLKKLV	*H. gertschi*
NDBP-5.7	5	C5J886	ILSAIWSGIKSLF-NH_2_	*O. cayaporum*
NDBP-5.8	5	C5J887	GILGKIWEGVKSLI	*O. cayaporum*
ToAcP	^∗∗^	LT576031	EEDDLLGFSEEDLKAIKEHRAKNA-NH_2_	*T. obscurus*


*^∗^Accession number recently requested; ^∗∗^subfamily not designated. NDBP, non-disulfide-bridged peptide.*

Comparative analyses of amino acids sequences were performed employing the Basic Local Alignment Search Tool (BLAST^1^) and Collection of Anti-Microbial Peptides (CAMP^2^) databases to identify peptides with at least 40% of identity to our queries sequence and to which antimicrobial activity was previously determined. The multiple sequence alignments were done using the M-Coffee tool^3^ on the sequences identified in the previous step, and the consensus sequences were represented as a sequence logo, generated at Weblogo web server^4^.

### Chemical Peptide Synthesis

Peptides were chemically synthesized using FMOC-Butila (AminoTech Pesquisa e Desenvolvimento LTDA, Brazil), and further purified by reverse-phase high performance liquid chromatography (HPLC). Peptides ToAP1, ToAP2S1, ToAP3, NDBP-4.23, ToAP4, and NDBP-5.7 were synthesized with C-terminal amidation. To assess the purity and sequence correctness, peptides were submitted to matrix-assisted laser desorption/ionization time of flight mass spectrometry (MALDI-TOF/TOF MS; UltraFlex III, Bruker Daltonics, Germany), under reflector (MS) and LIFT^TM^ (MS/MS) positive modes. Matrix solution was prepared using 5.0 mg of α-cyano-4-hydroxycinnamic acid (Sigma, USA) and solubilized with 250 μL of acetonitrile, 200 μL of deionized water, and with 50 μL of an aqueous trifluoroacetic acid solution (at 3% by volume). The aqueous solution of the peptide was mixed with a saturated matrix solution (1:3 peptide/matrix) and air-dried. The monoisotopic molecular mass of the ion corresponding to the peptide of interest was determined by the ratio between *m/z* peaks in the spread profile (*m/z* ratio from 600 to 3,000). Additionally, interpretation of the MS/MS mass spectra and *de novo* sequencing of peptides was performed using the FlexAnalysis 3.0 software (Bruker Daltonics, Germany). Throughout the experiment, the stability of each peptide was reevaluated using the same mass spectrometry parameters detailed above. Only samples containing more than 95% of the peptide (high purity) were used for biological experiments. Peptides were stored at -20°C and dissolved in Milli-Q water before each experiment.

### *In silico* Analysis of Putative Secondary Structure and Peptides Physicochemical Properties

The putative secondary structures and the α-helix content (%) were predicted using the software PHD as described previously (Guo et al., 2013)^5^. The hydrophobicity and the helical hydrophobic moment of the peptides were determined in the HeliQuest web server^6^. Other physicochemical properties were determined by ProtParam tool^7^ (Wilkins et al., 1999). Helical wheel projections were generated at the respective web server^8^.

### Fungal Strains and Growth Conditions

*Candida albicans* strain SC 5314 (ATCC MYA-2876) was provided by Dr. Joshua Nosanchuk (New York, NY, USA). *C. glabrata* ATCC 90030, *C. parapsilosis* ATCC 22019, and *C. tropicalis* ATCC 750 were provided by Dr. Érika Kioshima (Universidade Estadual de Maringá, Paraná, Brazil). *C. neoformans* var. *grubii* strain H99 (serotype A, ATCC 208821) was provided by Dr. John Perfect (Durham, NC, USA). *C. neoformans* var. *neoformans* B3501 (serotype D, ATCC 34873) was obtained from ATCC (Manassas, VA, USA). All strains were stored as frozen stocks in 35% glycerol at -80°C. Before each experiment, all *Candida* spp. strains were grown in Sabouraud’s dextrose broth overnight at 30°C with agitation. *C. neoformans* strains were grown in the same conditions for 24 h. Fungal cells were collected by centrifugation, washed three times in phosphate buffered saline (PBS) (137 mM NaCl, 2.7 mM KCl, 10 mM Na_2_HPO_4_, 2 mM KH_2_PO_4_) and inoculated in Roswell Park Memorial Institute (RPMI) 1640 medium supplemented with L-glutamine and buffered to pH 7.0 with 165 mM 3-(*N*-morpholino)propanesulfonic acid for experiments described below.

### Antifungal Assay

*In vitro* antifungal assays were performed according to broth microdilution susceptibility test from Clinical and Laboratory Standards Institute (CLSI) M27-A3 guidelines with some modifications. Briefly, twofold serial dilutions of each peptide were prepared in 96-well polystyrene microplates to a final volume of 50 μL. Amphotericin B (Amp B; A2942, Sigma-Aldrich), a known antifungal drug, was used as a positive control. Final concentrations of each peptide and Amp B ranged from 100 to 0.78 μM and 16 to 0.03 μg/mL, respectively, except in tests with *C. glabrata* and *C. parapsilosis*, where the final peptide concentration ranged from 400 to 0.78 μM. In each plate, wells were included without peptide as a growth control. Then, 50 μL of the adjusted inoculum in RPMI-1640 medium were added to each well to a final concentration of 2 × 10^3^ cells/mL for *Candida* spp. and 10^4^ cells/mL for *C. neoformans* strains. The plates were incubated at 37°C for 24 and 48 h, respectively. The strains of *C. neoformans* were also incubated with shaking (200 rpm). The minimum inhibitory concentration (MIC) was defined as the lowest AMP concentration that completely inhibited visible fungal growth at the end of the incubation period. The experiments were performed at least three times on separate dates.

### Hemolytic Assay

Considering the potential use of the AMPs as treatment against pathogens, even if in a more distant future, some of their properties against host cells should be addressed. To achieve this goal, we have performed hemolytic assay to evaluate if these molecules are toxic or harmless to red blood cells. Further, we have also tested the possible cytotoxicity of those peptides against other mammalian cell types by following the cell viability score after treatment. The solution of human red blood cells was treated with the peptide at levels ranging from 100 to 0.78 μM. Whole human blood was collected from healthy donors in vials with ethylenediaminetetraacetic acid (EDTA). The red blood cells were separated by centrifugation and resuspended in PBS. After the centrifugation (800 *g*, 5 min at 4 ± 1°C) the supernatant was removed and the red blood cells pellet was resuspended to a 3% hematocrit in sterile water (considered as 100% hemolysis) and PBS (taken as blank for spectrophotometric measurement). Fifty microliters of the 3% cell suspension in PBS were added to different final concentrations of the peptide (v/v) in a 96-well polystyrene microplate and then incubated for 1 h (Nahar et al., 2008; Zhao et al., 2009; Qi et al., 2010). The microplates were centrifuged and the supernatant was collected and analyzed at 540 nm using a microplate spectrophotometer. The percentage of hemolysis was calculated relative to the positive control as performed by Nahar et al. (2008). The procedures were approved by the Ethics Committee on Human Research of Faculty of Medicine/University of Brasília (UnB) and conform to FDA standards (UnBDoc no. 66704/2016).

### Cytotoxic Test Using Peritoneal Macrophages

BALB/c mice were injected with thioglycolate 72 h before peritoneal macrophages were harvested. They were diluted to a concentration of 10^6^ cell/mL in RPMI-1640 supplemented with penicillin/streptomycin (100 U/mL, 100 g/mL), 2 mM L-glutamine, 2 mM non-essential amino acids, 1 mM sodium pyruvate (all reagents from Sigma-Aldrich, St Louis, MO, USA) and 10% fetal bovine serum (FBS), and incubated in 96-well polystyrene microplates at 37°C, 5% CO_2_ for 24 h. The peptides were added then and cells were further incubated for 24 h as described previously (Cruz et al., 2005). Three independent experiments were performed. Cytotoxic activity was evaluated by the release of cytoplasmic enzyme lactate dehydrogenase due to cell lysis using the Cytotox kit (Promega). Briefly, at the end of the incubation period, supernatants were collected, the adhered cells were washed and the toxicity was determined according to the manufacturer’s protocol. The procedures were approved by the Ethics Committee on Animal Research of Institute of Biology (UnBDOC 52657/2011)/University of Brasília (UnB).

### Effects of Peptides on *Candida albicans* Biofilms

The effects of the peptides NDBP-4.23, ToAP1, ToAP2, and NDBP-5.7 on *C. albicans* biofilms were analyzed according to the protocol described by Pierce et al. (2008). Briefly, *C. albicans* SC 5314 (10^6^ cells/mL) was grown on 96-well polystyrene microplates and 4 h after seeding the peptides or Amp B were added to assess their effects on the first step of biofilm formation, the initial cell adherence. After 24 h of treatment, biofilm formation was measured by the metabolic assay based on the reduction of 2,3-bis-(2-methoxy-4-nitro-5-sulfophenyl)-2H-tetrazolium-5-carboxanilide (XTT). For the purpose of investigating effects of peptides on mature biofilms, the *Candida* cells were allowed to grow for 24 h prior to addition of the peptides or Amp B and the XTT assay was performed in the same way. All results were analyzed for statistical significance using analysis of variance (ANOVA) followed by Tukey post-test using GraphPad Prism 6 and expressed in terms of biofilm viability as XTT-readings percentage normalized by the control groups. Each compound was tested in triplicate and the experiments were performed at least twice on separate dates.

### Circular Dichroism

Circular dichroism (CD) assays were carried out using Jasco J-815 spectropolarimeter equipped with a Peltier-type temperature controller. Peptides (0.2 mg/mL) were analyzed in water at 37°C in absence or the presence of trifluoroethanol (TFE) (10, 30, and 50%). The mean spectrum of three consecutive experiments was corrected for the baseline buffer contribution. The observed ellipticities were converted into molar ellipticity ([θ]) based on molecular mass per residue of 115 Da (Adler et al., 1973). The α helix secondary structure content (*f*θ) was estimated considering the values of [θ]_208 nm_ as a function of TFE concentration using the following equation (Greenfield and Fasman, 1969):

$$f_{H}=\frac{\left({\left[\theta \right]}_{208}-4000\right)}{\left(-33000-4000\right)}$$

## Results

### *In silico* Prediction of Major Physicochemical Properties and Secondary Structure of Peptides

**FIGURE 1:**
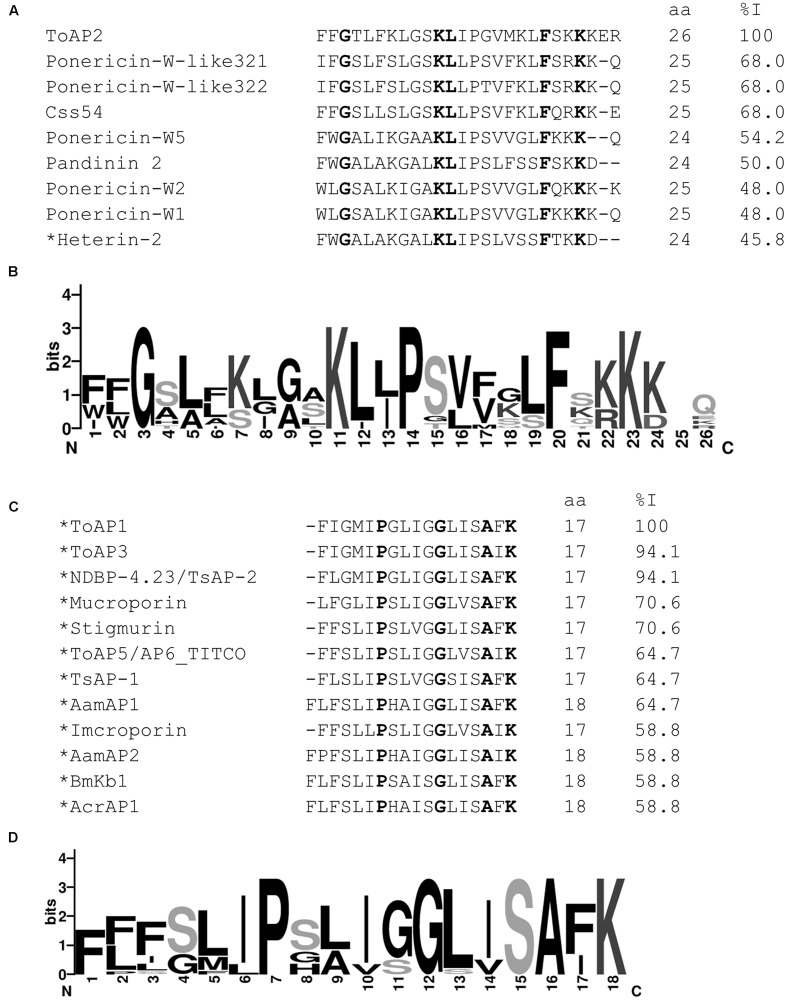
**Comparative analysis of ToAP2, ToAP1, ToAP3, ToAP4 against previously described AMPs.** Conserved amino acid residues are marked bold. Asterisks indicate peptides with amidated C-termini. **(A,B)** Multiple sequence alignment of ToAP2 mature peptide with known AMPs from scorpion and ant venoms such as Ponericin-W-like 32.1 [UniProt: P0CI91], Ponericin-W-like 32.2 [UniProt: P0CI92], Css54 [UniProt: P0DL41], Pandinin 2 [UniProt: P83240], Heterin-2 [UniProt: A0A0C4G5K0], Ponericin-W1 [UniProt: P82423], Ponericin-W2 [UniProt: P82424], and Ponericin-W5 [UniProt: P82427] and their consensus sequence logo. **(C,D)** Multiple sequence alignment of ToAP1 mature peptide with known antimicrobial peptides from scorpion venom such as NDBP-4.23 [UniProt: Q5G8B5/S6D3A7], Mucroporin [UniProt: B9UIY3], Imcroporin [UniProt: C7B247], Stigmurin [GenBank: JK483709], AP6_TITCO [UniProt: Q5G8B3], TsAP-1 [UniProt: S6CWV8], AamAP1 [UniProt: G8YYA5], AamAP2 [UniProt: G8YYA6], BmKb1 [UniProt: Q718F4], and AcrAP1 [UniProt: A0A0A1I6E7] and their consensus sequence logo.

The primary structures of ToAP2, ToAP1, ToAP3, ToAP4, and ToAcP peptides from *T. obscurus* were used as queries on BLAST. Data analysis revealed that ToAP2 is similar to peptides previously described as belonging to NDBP subfamily 3 and to AMPs from ant venom (**Figures 1A,B**). ToAP1, ToAP3, and ToAP4 showed similarity to AMPs belonging to NDBP subfamily 4 (**Figures 1C,D**). ToAP4 also has 100% identity to putative AMP clone 6 from *T. costatus* [UniProt: Q5G8B3]. ToAcP did not show similarity to any AMPs described before and shares 90% identity to Toxin Tp3 [UniProt: P0DL22], a peptide from *Tityus pachyurus* with no toxicity to mammalian cells and without known biological activity (Barona et al., 2006). The result of these analyses indicates that peptide ToAP2 belong to NDBP subfamily 3 and ToAP1, ToAP3, ToAP4 belongs to NDBP subfamily 4.

**FIGURE 2:**
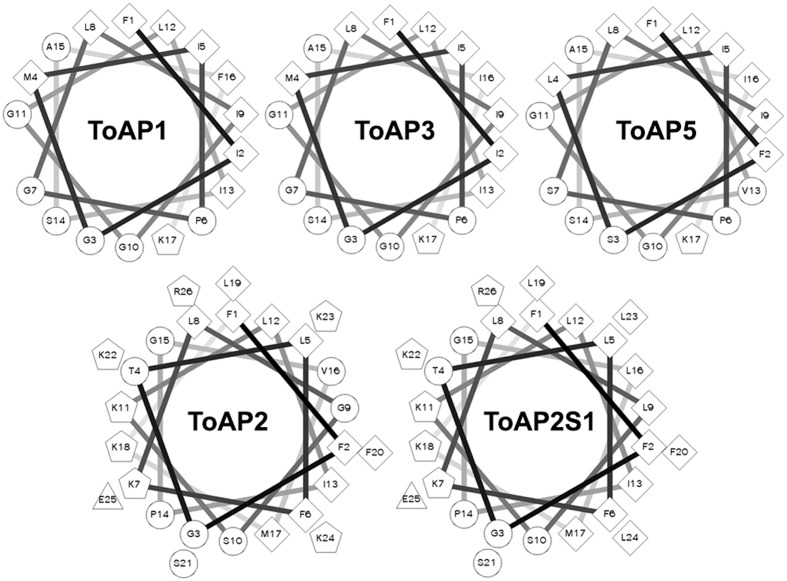
**Helical wheel projections of the α-helical structured peptides.** The projections were generated at the web server for this tool (http://rzlab.ucr.edu/scripts/wheel/wheel.cgi). Diamonds, hydrophobic residues; circles, hydrophilic residues, non-charged; pentagons, positively charged residues; triangles, negatively charged residues.

The results of *in silico* secondary structure and physicochemical property prediction for the peptides are summarized in **Table 2**. Except for ToAP2 and ToAcP, which have net charges at pH 7.0 of +6 and -4, respectively, all others showed a net charge of +1. All peptides have a predicted helical content of at least 53.85% and, except for ToAcP, had positive hydrophobicity values, which means that in theory they are slightly hydrophobic. ToAP2S1, an analog from ToAP2, had different physicochemical parameters. Its net charge was reduced from +6 to +4 as a result of the substitution of two lysines for leucines; and both its hydrophobicity and hydrophobic moment were increased. ToAP2S1 also had its predicted helical content increased from 57.69 to 88.46% relative to its prototype ToAP2. Helical wheel projections predict that ToAP2, ToAP2S1, ToAP1, ToAP3, and ToAP4, once adopting a helical conformation, are amphipathic peptides, thus presenting distinct hydrophobic and hydrophilic faces (**Figure 2**). ToAcP, however, presents only a hydrophilic face. The lack of a distinct hydrophobic face compromises its amphipathicity.

**Table 2:** Peptide sequences, putative secondary structure, and physicochemical properties.

Peptide	Sequence and secondary structure	Length	α-helix (%)	Hydrophobicity	Hydrophobic moment	Net charge
ToAP2	FFGTLFKLGSKLIPGVMKLFSKKKER	26	57.69	0.443	0.460	+6
	ccchhhhcccchhhhhhhhhhhcccc					
ToAP2S1	FFGTLFKLLSKLIPGLMKLFSKLLER-NH_2_	26	88.46	0.734	0.672	+4
	cchhhhhhhhhhhhhhhhhhhhhhhc					
Con10	FWSFLVKAASKILPSLIGGGDDNKSSS	27	59.26	0.435	0.331	+1
	chhhhhhhhhhhhhhhhcccccccccc					
ToAP1	FIGMIPGLIGGLISAFK-NH_2_	17	64.71	0.906	0.597	+1
	ccchhhhhhhhhhhccc					
ToAP3	FIGMIPGLIGGLISAIK-NH2	17	64.71	0.907	0.597	+1
	cccchhhhhhhhhhhcc					
NDBP-4.23	FLGMIPGLIGGLISAFK-NH_2_	17	76.47	0.908	0.594	+1
	ccchhhhhhhhhhhhhc					
ToAP4	FFSLIPSLIGGLVSAIK-NH2	17	70.59	0.895	0.592	+1
	cccchhhhhhhhhhhhc					
NDBP-5.6	FIFDLLKKLV	10	70.00	0.895	0.715	+1
	chhhhhhhcc					
NDBP-5.7	ILSAIWSGIKSLF-NH_2_	13	53.85	0.926	0.668	+1
	ccchhhhchhhcc					
NDBP-5.8	GILGKIWEGVKSLI	14	85.71	0.686	0.680	+1
	chhhhhhhhhhhhc					
ToAcP	EEDDLLGFSEEDLKAIKEHRAKNA-NH_2_	24	54.17	-0.016	0.248	-4
	ccccccccchhhhhhhhhhhhhcc					


*NDBP, non-disulfide-bridged peptide; the letter “c” represents random coil and “h” represents α-helix; the putative secondary structures and the α-helix content (%) were predicted using the software PHD (Guo et al., 2013); the hydrophobicity and the helical hydrophobic moment of the peptides were determined in the HeliQuest web server; length and net charge properties were determined by ProtParam tool.*

### Antimicrobial Assay

Antifungal assays were performed against different species of *Candida* and *C. neoformans* (**Table 3**).

**Table 3:** Inhibitory concentrations of different peptides against *Candida* spp. and two *Cryptococcus neoformans* strains.

Peptide	Minimum inhibitory concentration (μM)
	
	*C. albicans* (SC5314)	*C. tropicalis* (ATCC 750)	*C. parapsilosis* (ATCC 22019)	*C. glabrata* (ATCC 90030)	*C. neoformans* (H99 serotype A)	*C. neoformans* (B3501 serotype D)
ToAP2	12.5	3.12	50	200	12.5	6.25
Con10	100	12.5	200	200	50	25
ToAP3	25	12.5	100	>400	100	25
NDBP-4.23	50	6.25	100	>400	25	12.5
ToAP1	50	12.5	200	>400	25	12.5
NDBP-5.7	25	25	>400	>400	25	12.5
NDBP-5.8	100	25	200	>400	100	50
Amp B	1.08	2.16	2.16	1.08	0.27	0.135


*NDBP, non-disulfide-bridged peptide; Amp B, amphotericin B. The values of 0.135, 0.27, 1.08 and 2,16 μM of Amp B are equivalent to 0.125, 0,25, 1 and 2 μg/mL of Amp B respectively.*

Seven peptides were active against *Candida* spp. and *C. neoformans*. ToAP2 and Con10, both from NDBP subfamily 3, had antifungal activity against all strains tested with MICs values ranging from 3.12 to 200 μM and 12.5 to 200 μM, respectively. Among peptides from NDBP subfamily 4, only ToAP4 has not shown antifungal activity in the tested concentrations (data not shown). The other peptides from this subfamily presented antimicrobial activity against all *Candida* spp. and *C. neoformans* in the same MIC range (6.25–200 μM), except for *C. glabrata*, which is insensitive to them. *C. glabrata* is also resistant to all peptides from NDBP subfamily 5. Among those subfamily peptides, NDBP 5.7 presented antifungal activity with MIC values ranging from 12.5 to 25 μM and NDBP 5.8 inhibited the growth of pathogens tested at concentrations ranging from 50 to 200 μM. NDBP-5.6 and ToAcP did not present antifungal activity in this assay (data not shown).

Since ToAP2 showed the lower MIC against all pathogens tested in this work, derivatives of its structure were produced in order to increase the α-helical content. The modified peptide, ToAP2S1 (**Table 1**) was synthesized and tested against two *Candida* species (*C. albicans* and *C. glabrata*) and *C. neoformans* H99. ToAP2S1 did not show antifungal activity in the tested concentrations (data not shown), demonstrating that the changes in peptide sequence abolished antifungal activity relative to the reference peptide.

Based on results from antimicrobial assay, we decided to proceed only with those peptides (at least one per family) that showed best antimicrobial activity.

### Hemolysis and Cytotoxicity Assays

**FIGURE 3:**
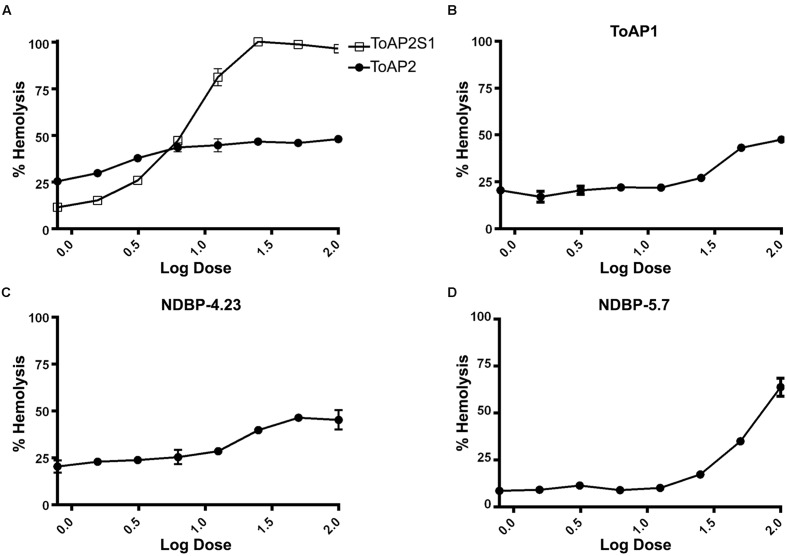
**Hemolytic activity of peptides against human erythrocytes.** **(A)** ToAP2 and ToAP2S1, **(B)** ToAP1, **(C)** NDBP-4.23, and **(D)** NDBP-5.7.

The hemolytic activities of peptides ToAP2, NDBP-4.23, ToAP1, and NDBP-5.7 were evaluated against human erythrocytes (**Figure 3**). ToAP2S1 was also tested to evaluate if it also has lost its hemolytic activity. NDBP-4.23 and ToAP1 might be considered of interest to future studies, because in all the tested concentrations their hemolysis curves were significantly lower than 50% of hemolysis (Conlon et al., 2007; Thaker et al., 2011; **Figures 3B,C**). However, NDBP-5.7 presents a hemolysis percent higher than 50% in concentrations up to 50 μM (**Figure 3D**). Comparing the original peptide ToAP2 and the modified ToAP2S1, the last one shows a higher hemolysis curve with concentrations up to 25 μM while the ToAP2 maintained its curve above 50% of hemolysis (**Figure 3A**).

**FIGURE 4:**
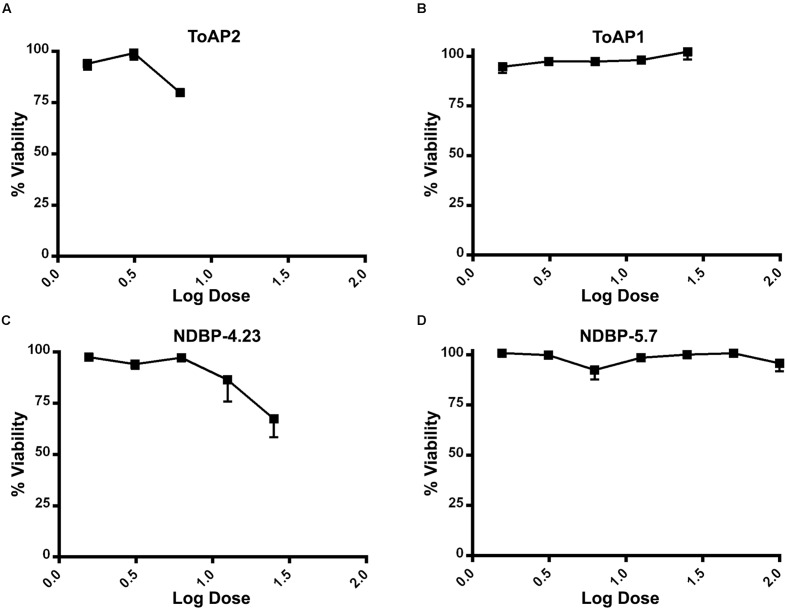
**Cytotoxicity of peptides against peritoneal macrophages of BALB/c mice.** **(A)** ToAP2, **(B)** ToAP1, **(C)** NDBP-4.23, and **(D)** NDBP-5.7.

Toxicity to mammalian cells was further evaluated by assessing the viability of murine peritoneal macrophages in the presence of the peptides (**Figure 4**). The threshold of acceptable cytotoxicity was set at 50% viability as postulated elsewhere (Thaker et al., 2011; Zhao et al., 2012). In conformity with the hemolytic test, ToAP2 and NDBP-4.23 in concentrations below 6.25 and 25 μM, respectively, have shown low cytotoxicity (**Figures 4A–C**). For ToAP1 and NDBP-5.7, peritoneal macrophages displayed higher viability in the whole range of peptide concentrations (**Figures 4B–D**). The difference in the hemolytic curves between ToAP2 and its analog (ToAP2S1) could be explained by the higher hydrophobicity of ToAP2S1 (**Table 2**) and consequent tendency to self-associate. Chen et al. (2007) demonstrated that there is an optimal hydrophobicity window for AMPs, below which antimicrobial activity can be lost and above which self-association becomes highly likely. Peptide aggregation prevents penetration through the capsule and cell wall of microorganisms (Jiang et al., 2008). However, the higher hydrophobicity does not affect their activity on the eukaryotic membrane (Chen et al., 2007), which explains the lower antifungal activity and the higher hemolytic activity of the modified ToAP2S1, respectively.

### Effects of the Peptides on *Candida albicans* Biofilms

We visualized *C. albicans* biofilms at 4 and 24 h by fluorescence microscopy using uvitex 2B, which binds chitin in the fungal cell wall, and they exhibited typical features of mature biofilms, including a basal layer of yeast cells and a thicker layer of yeast and hyphae cells (Supplementary Material). This structure was very similar to the morphology of *C. albicans* biofilms presented in other works in the literature (Pierce et al., 2014, 2015). Additionally, the inhibition values of biofilms by Amp B were similar to those found by Ramage et al. (2001, 2002).

**FIGURE 5:**
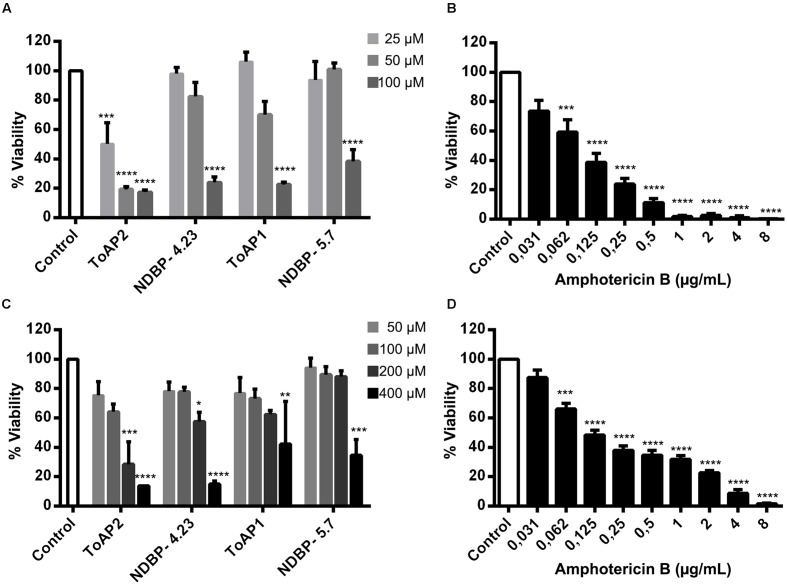
**Effects of the peptides ToAP2, NDBP-4.23, ToAP1, NDBP-5.7, and amphotericin B on *C. albicans* biofilm formation.** Effects of the peptides **(A)** and Amp B **(B)** on the initial adherence and effects of the peptides **(C)** and Amp B **(D)** on the mature stage. Results are expressed in terms of biofilm viability as XTT-reduction assay readings percentage, normalized by the control groups. The experiments were performed at least twice on different days. Statistical analyses: ANOVA and Turkey post-test (^∗∗∗∗^*P* ≤ 0.0001; ^∗∗∗^*P* ≤ 0.001; ^∗∗^*P* ≤ 0.01; ^∗^*P* ≤ 0.05). Mean ± SEM.

The effects of ToAP2, NDBP-4.23, ToAP1, and NDBP-5.7 on *C. albicans* biofilms were evaluated on the initial adherence step (**Figure 5A**) and on the mature biofilm (**Figure 5C**). As a control, the effect of Amp B was also shown at both time points (**Figures 5B,D**), with a reduction of at least 80% in biofilm viability at 0.25 μg/mL for the initial adherence and 2 μg/mL for the three-dimensional architecture. All the peptides were active against biofilm formation, albeit at a higher concentration compared to what was observed against planktonic cells (100 μM for the initial adherence and 400 μM for the three-dimensional architecture). ToAP2 was the most active peptide at the two different stages of biofilm formation, with a lower effective concentration than the others (25 μM for the initial adherence and 200 μM for the three-dimensional architecture).

### Circular Dichroism

**FIGURE 6:**
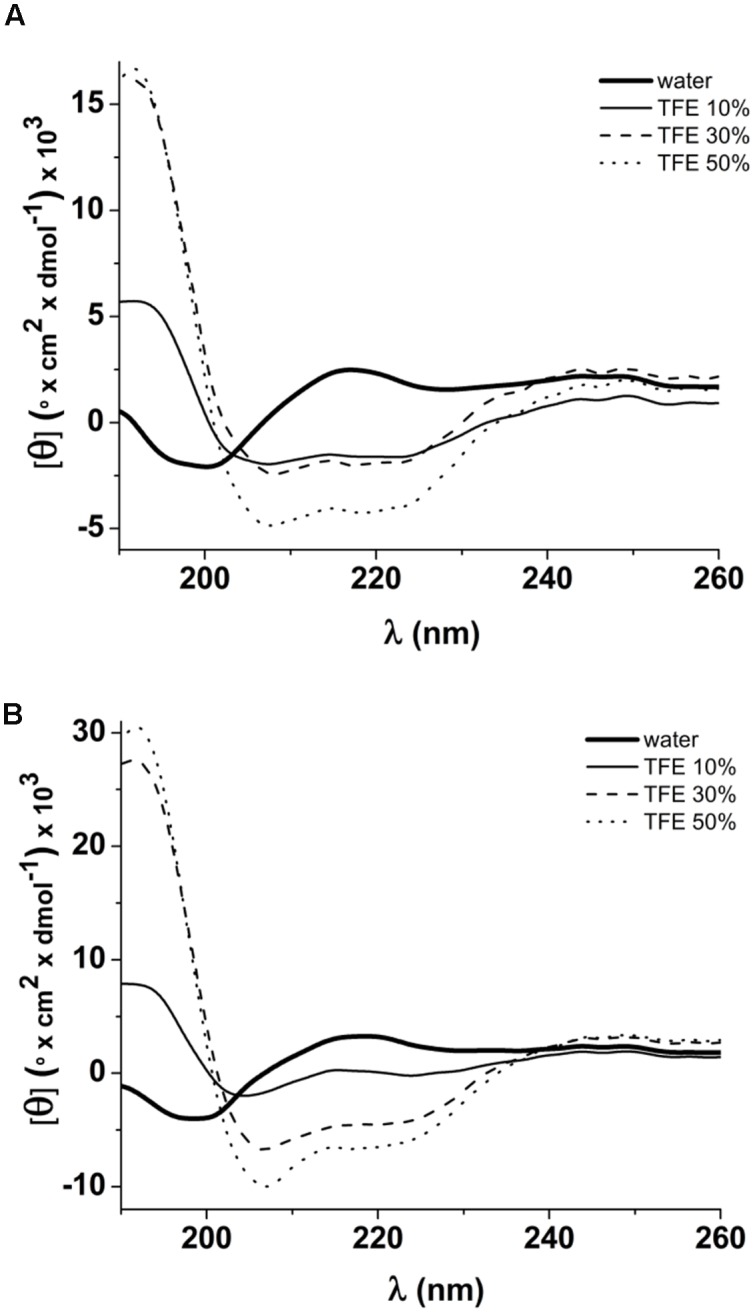
**FAR-UV CD spectra of peptides in water and 10, 30, 50% TFE.** **(A)** Spectra of ToAP2 presenting a red shift from 200 nm to 208 and 222 nm in the presence of TFE; **(B)** Spectra of ToAP2S1 showing an increase of the dichroic signals at 208 and 222 nm from water to TFE.

The secondary structures of native (ToAP2) and modified (ToAP2S1) peptides were assessed by CD spectroscopy. FAR-UV CD spectra of ToAP2 in water at 37°C revealed a typical disordered structural pattern (**Figure 6A**). At increasing concentrations of 10, 30, and 50% TFE (v/v), a red shift from 200 to 208 and 222 nm and increased negative dichroic signals were observed (**Figure 6A**). These results show that ToAP2 lacks secondary structure in aqueous environments and has a tendency to adopt an α-helical pattern at aqueous environments in the present of the co-solvent TFE (**Table 4**). In contrast, an α-helical conformation was observed for the modified peptide ToAP2S1 in water at 37°C even in the absence of TFE. In the presence of 10, 30, and 50% TFE, intense negative dichroic bands at 208 and 222 nm were observed (**Figure 6B**), which are compatible with higher α-helix content (**Table 4**). Fractional helicity of peptides in water and TFE environments was calculated considering the molar ellipticity at 208 nm (**Table 4**). There was an increased α-helix content for both peptides as a function of TFE, with ToAP2S1 presenting approximately 17% of α-helix in water in the absence of the co-solvent.

**Table 4:** α-Helix content (%) of peptides in water, and TFE at 10, 30, and 50%.

Solvent	α-Helix content (%) of peptides	
	
	ToAP2	ToAP2S1
Water	9.8	16.5
TFE 10%	12.9	22.9
TFE 30%	20.8	24.3
TFE 50%	25.8	24.7


## Discussion

We have witnessed a steady increase in the incidence of systemic fungal diseases in the last decades, partially due to medical advances and the rise of immunosuppressive diseases. Although antifungal therapy is somewhat efficient, there are only four classes of agents, many of which have significant side effects. In addition, there is an increasing number of fungal species with natural or acquired resistance to these drugs. For instance, the most recent class of antifungals, the echinocandins, was discovered in the seventies and only became available for clinical therapy in 2002. Since then, the search for new antifungal targets and potential drugs for more efficient and less toxic treatments has been an ongoing work. AMPs are attractive alternatives as antifungal drugs and here we describe seven scorpion venom-derived peptides with promising antimicrobial activity against *Candida* spp. and/or *C. neoformans* strains, all of which are major fungal pathogens.

Regardless of the strain, ToAP2, the most cationic peptide, showed the lowest inhibitory concentrations in our tests. ToAP2 and Con10 exhibited sequence similarity to Pandinin 2, for which anti-*C. albicans* activity has been described with a MIC of 19.1 μM (Corzo et al., 2001). In contrast, while ToAP2 presented MIC values around this range, Con10 showed much higher values for *C. albicans* and most of the other tested strains. Despite the similarity, Con10 has lower net positive charge (+1) than Pandinin 2 (+3). Conversely, ToAP2 physicochemical properties are more similar to those of Pandinin 2: its net positive charge is +6. Several studies have shown that analog of AMPs with amino acids substitutions to increase positive net charge had better antifungal activity (Nikawa et al., 2004), which may explain the poor antifungal activity of Con10 relative to ToAP2 and Pandinin 2.

We also analyzed the secondary structures of the peptides in water and in a mimetic membrane environment (aqueous co-solvent TFE) by CD spectroscopy. The FAR-UV CD spectra showed that ToAP2 and its modified version ToAP2S1 have typical disordered and α-helical structures in water at 37°C, respectively. There was an increase in α-helix content for both peptides as a function of TFE concentration (**Table 4**), indicating the potential of these molecules to interact with the cellular membrane, a classical feature of α-helical AMPs. Stability assays also showed that the non-modified peptide presented lower α-helix content and lower stability than the modified peptide. These observations may indicate that net charge is an important parameter for antifungal activity as well as to the conformation changes induced by the interaction with the fungal membrane.

ToAP1 and ToAP3 share similarity with AamAP1, AamAP2, and AcrAP1 (**Figure 1**), peptides that have already been documented as having anti-*C. albicans* activity (Almaaytah et al., 2012; Du et al., 2014). Interestingly, ToAP1 and ToAP3 only differ at one residue at position 16, respectively a Phe and an Ile. While ToAP3 has better anti-*Candida* spp. activity, ToAP1 showed better activity against *C. neoformans* (**Table 3**). This result suggests that Phe16 in ToAP1 could be essential for anti-*C. neoformans* activity. The lack of activity of ToAP4 against the fungal strains tested in our assay condition is worth noting, in spite of its 82.3% identity with Stigmurin (**Figure 1**), a peptide from *Tityus stigmurus* that inhibits the growth of *C. albicans* and *C. glabrata* with MIC values of 34.8 μM and 69.5 μM, respectively (de Melo et al., 2015). This difference could be due to different methods used to evaluate antifungal activity or the difference in sequence itself. More studies are needed to evaluate this.

NDBP-5.7 and NDBP-5.8 showed high similarity to IsCT, IsCT2, and Patinin3, peptides with antifungal activity against *C. albicans* and *C. tropicalis* (Dai et al., 2002; Zeng et al., 2013). Considering that the C-terminal amidation modifies the net positive charge and stabilizes peptide structure, improving antimicrobial activity (Huang et al., 2010), the lack of this chemical group may explain the lower activity of NDBP-5.8 against the tested microorganisms (**Tables 2** and **3**). By its turn, the lack of antifungal activity of ToAcP probably could be explained by its net negative charge, which causes electrostatic repulsion from the microbial plasma membrane (van der Weerden et al., 2013).

Among the tested *Candida* species, *C. glabrata* was the most resistant, with inhibitory concentrations higher than 400 μM for all peptides. This result is in accordance with the literature describing the high intrinsic resistance shown by this species to most antifungal drugs, as well as to Histatin 5, one of the best candidacidal peptides already described (Sobel, 2006; Tati et al., 2013; Rodrigues et al., 2014). Conversely, *C. tropicalis* was the most susceptible organism among *Candida* species. As shown in **Table 3**, the AMPs exhibited their lowest inhibitory concentrations against *C. tropicalis*, in contrast with its described high virulence, associated infection mortality rates, and resistance to amphotericin B (CLSI M27-A3 protocol (Colombo et al., 2007). Consequently, the peptides tested in this work are promising candidates for the development of treatments for *C. tropicalis* infection.

With regard to the effects of AMPs against *C. neoformans*, the B3501 strain (serotype D) was more sensitive to the scorpion peptides than the H99 strain (serotype A). This is in accordance with the previously described higher susceptibility of this serotype to amphotericin B (Thompson et al., 2009). *C. neoformans* has a negatively charged polysaccharide capsule that is one of the most important virulence factors of this yeast (McFadden et al., 2006). Its thickness varies according to strain, with strain B3501 presenting a thicker capsule than H99 under non-inducing conditions. Therefore, it is possible that the capsule exerts an electrostatic attraction effect on cationic AMPs, which may explain the higher susceptibility of strain B3501. Similarly, susceptibility of *C. neoformans* to polymyxin B was previously reported to be dependent on fungal capsule thickness (Zhai and Lin, 2013).

In addition to the effects on planktonic cells described above, we also analyzed their action against *C. albicans* biofilms. Biofilms represent a great health concern due to their intrinsic resistance to antimicrobial treatment and to the patient immune response. The resistance involves different factors like overexpression of drug resistance genes and impairment of drug penetration due the thickness of the extracellular matrix. All peptides tested (ToAP2, NDBP-4.23, ToAP1, and NDBP-5.7) reduced biofilm on both the initial cell adherence stage and the mature phase, albeit at higher concentration ranges relative to planktonic cells, which holds true for all antimicrobials. Again, ToAP2 was the most active peptide at the two different stages of biofilm formation, presenting a lower effective concentration than the other peptides (25 μM for the initial adherence and 200 μM for the three-dimensional architecture). These results are encouraging, since the peptides showed an antifungal effect in different phases of the biofilm process with lower concentrations than expected for prospective antifungals (100–1,000 times higher than concentrations used against planktonic cells (Pierce et al., 2008). Furthermore, hemolysis and cytotoxicity assays showed promising results regarding the selective toxicity of the tested peptides. Most of the tested peptides can be considered of interest for future studies, as they caused hemolysis of less than 50% of erythrocytes and more than 50% of murine peritoneal macrophages remained viable in their presence.

In summary, we described seven scorpion venom-derived AMPs with antifungal activity against cells of *Candida* spp. and *C. neoformans* and against *C. albicans* biofilms, including two novel molecules. This is the first report of scorpion-derived AMPs against *C. neoformans* and our results underscore the potential of scorpion-venom as a source of antimicrobials. The observed effects of the described peptides during and after *C. albicans* biofilm formation highlight the antimicrobial potential of these molecules in the face of the huge impact of biofilm-derived infections in hospitalized patients. Further characterization of their mechanisms of action and interaction with available antifungals, followed by molecular optimization to reduce their toxicity to host cells and increase antimicrobial activity are need to fully clarify their real potential as antifungals.

## Author Contributions

FG and NV designed and carried out all AMP screening experiments and characterization of their antifungal properties. KS, MR, PC, AT, and AB designed and carried out the hemolysis and cytotoxicity assays. AA and SF contributed to CD experiments. OF contributed to MS experiments. MO, NV, FG, PA, IS-P, and LD contributed in the biofilm assays. AN supervised the biofilm microscopy analysis. ES selected the AMPs to be used in this work, contributed to chemical peptide synthesis and design of modified peptides. MM carried out peptide sequence, purity, and stability assessments (MS assays), contributed to antimicrobial assays and oversaw statistical analysis. PA and IS-P supervised all work. FG, NV, KS, AB, SF, PA, and IS-P contributed in the writing and revision of the manuscript. LD, OF, ES, and MM also contributed to the final revision of the manuscript. All authors read and approved the final version of the manuscript.

## Conflict of Interest Statement

The authors declare that the research was conducted in the absence of any commercial or financial relationships that could be construed as a potential conflict of interest.

## Footnotes

1 http://blast.ncbi.nlm.nih.gov/Blast.cgi

2 http://www.camp.bicnirrh.res.in/

3 http://tcoffee.crg.cat/apps/tcoffee/do:mcoffee

4 http://weblogo.berkeley.edu/logo.cgi

5 https://npsa-prabi.ibcp.fr/cgi-bin/npsa_automat.pl?page=/NPSA/npsa_phd.html

6 http://heliquest.ipmc.cnrs.fr/cgi-bin/ComputParamsV2.py

7 http://web.expasy.org/protparam

8 http://rzlab.ucr.edu/scripts/wheel/wheel.cgi
