# Supplementary material for: Activity of Scorpion Venom-Derived Antifungal Peptides against Planktonic Cells of Candida spp. and Cryptococcus neoformans and Candida albicans Biofilms
Source: Front Microbiol. 2016 Nov 18;7:1844. doi: 10.3389/fmicb.2016.01844 (PMC5114273; doi:10.3389/fmicb.2016.01844)
Supplement: Supplementary file 1 [file Presentation_1.PDF]

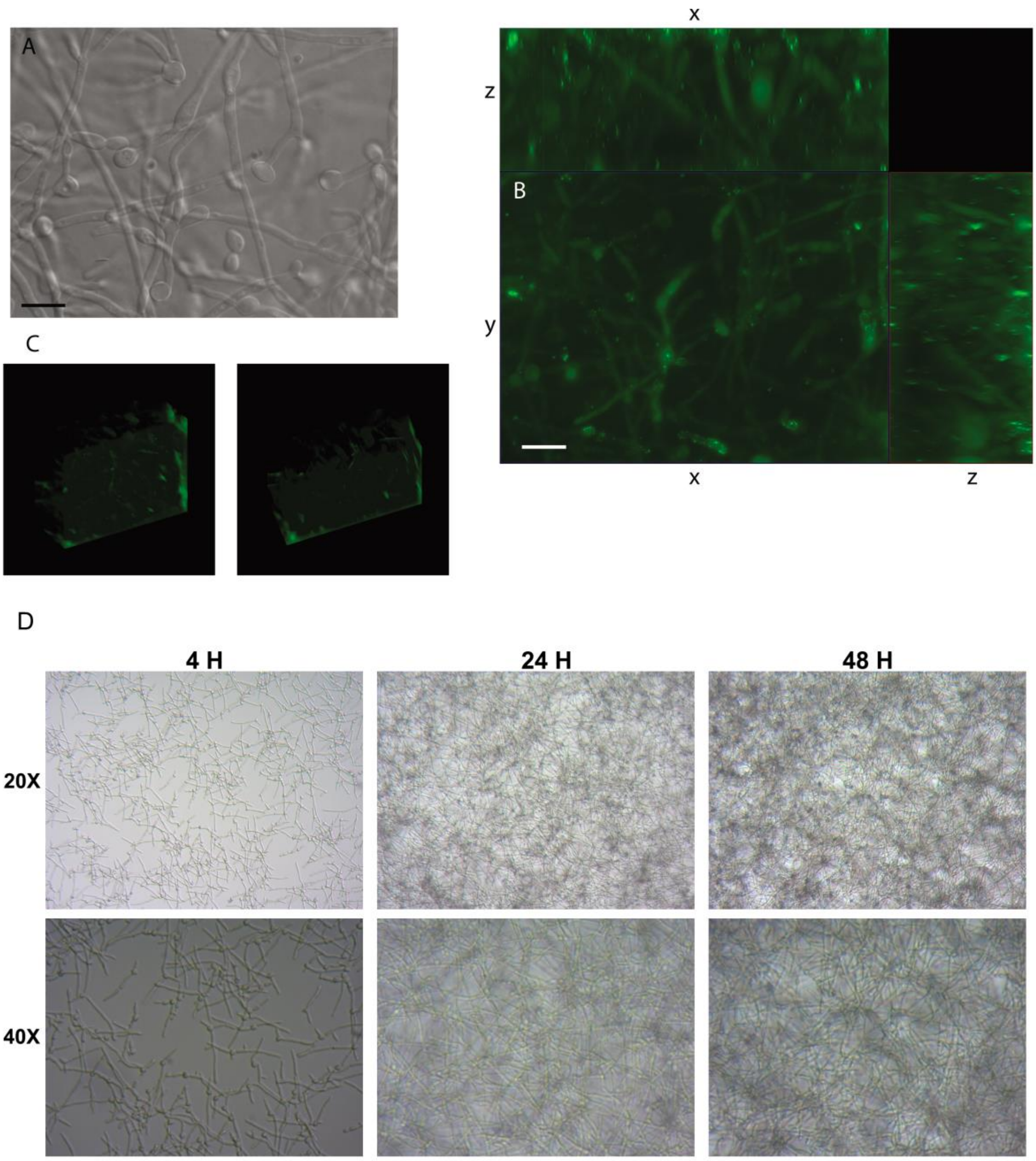

Figure 1R. Microscopic morphology of the *C. albicans* biofilm

C. albicans yeast cells were inoculated into a 13 mm round sterile coverslip inside a well of a 24-well plate. The cells were re-suspended in 1 mL of RPMI medium at the density of 10^6 cells/mL, conditions that are similar to those used to make biofilms for antimicrobial peptide testing. After 24h of incubation, the coverslips were washed 3 times with PBS to remove non-adherent cells and stained for 60 minutes with 100 µg/mL Uvitex 2B, a fluorescent probe that binds to chitin in the cell wall and extracellular matrix. The coverslips were then mounted in mounting medium SlowFade (Molecular Probes) and observed in a Zeiss Axio Observer Z1 inverted microscope equipped with a 63X NA 1.4 oil immersion objective and a CCD camera. Panel A – Differential interference contrast (DIC) image collected at the region of the biofilm closest to the coverslip, showing a basal layer of yeast cells from which hyphae that form the biofilm emerge. Panel B – Three dimensional projections of the stack of epifluorescence images collected from the ~30 µm thick biofilm. Notice the diffuse haze between the *C. albicans* cells,

which indicates chitin in the extracellular matrix. The image was based on a Z-stack of images 0,24 µm apart that were submitted to constrained iterative deconvolution. Panel C – Three-dimensional reconstructions of the biofilm, showing diffuse extracellular matrix obscuring the *C. albicans* biofilm, from which some hyphae protrude. All images were collected with the Zeiss ZEN software. ImageJ, VOXX 2 and Adobe Photoshop were used to manipulate the images. Scale bars – 10 µm. Panel D - C.albicans SC5314 biofilm morphology analyzed by light microscopy after 4, 24 and 48 hour of incubation.

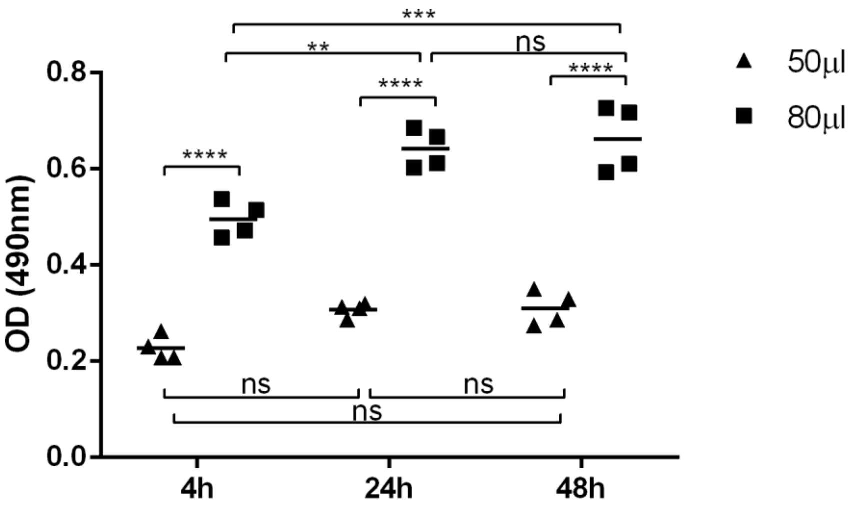

Figure 2R. Difference between supernatant volume used for OD 490nm reading (50 and 80µL) using XTT reduce assay. The experiments were performed four times independently. Statistical analyses: two-way ANOVA test and Turkey pos-test (**** P ≤ 0,0001; *** P ≤ 0,001; ** P ≤ 0,01; * P ≤ 0,05).

Table 1R. Sessile Minimum Inhibitory Concentration 50% ($SMIC_{50}$) of C. albicans SC5314 biofilm in response to Amphotericin B serially diluted from 0,01562 to 16 µg/mL.

	**$SMIC_{50}$ [ug/mL] (R^2)**		
	**4h**	**24h**	**48h**
**50 µl**	0,08420 (R^2 0,80)	0,1919 (R^2 0,86)	0,2857 (R^2 0,85)
**80 µl**	0,06812 (R^2 0,82)	0,1732 (R^2 0,92)	0,2424 (R^2 0,83)
